# Supplementary material for: Humoral Immunity of Unvaccinated COVID-19 Recovered vs. Naïve BNT162b2 Vaccinated Individuals: A Prospective Longitudinal Study
Source: Microorganisms. 2023 Jun 22;11(7):1628. doi: 10.3390/microorganisms11071628 (PMC10384358; doi:10.3390/microorganisms11071628)
Supplement: Supplementary file 1 [file microorganisms-11-01628-s001.zip › microorganisms-2413870-supplementary.pdf]

Supplementary Materials:

Table of Contents:		Page #
Table S1	Epidemiological and medical questionnaire	2
Table S2	Medical follow-up questionnaire	4
Table S3	COVID-19 disease severity	5
Table S4	Mixed Linear Models adjusted for sex and number of comorbidities, by period, for vaccinated versus recovered subjects (Outcome of log RBD - binding IgG)	5
Table S5	Mixed Linear Models adjusted for sex and number of comorbidities, by period, for vaccinated versus recovered subjects (Outcome of log NAbs)	5
Table S6	Mixed linear regression model for calculating the difference between recovered and vaccinated subjects in two age groups (Outcome of log RBD - binding IgG)	6
Table S7	Mixed linear regression model for calculating the difference between recovered and vaccinated subjects in two BMI groups (Outcome of log RBD - binding IgG)	6
Table S8	Mixed linear regression model for calculating the difference between recovered and vaccinated subjects in females and males (Outcome of log RBD - binding IgG).	7
Table S9	Mixed linear regression model for calculating the difference between recovered and vaccinated subjects in two age groups (Outcome of log NAbs).	7
Table S10	Mixed linear regression model for calculating the difference between recovered and vaccinated subjects in two BMI groups (Outcome of log NAbs).	8
Table S11	Mixed linear regression model for calculating the difference between recovered and vaccinated subjects in females and males (Outcome of log NAbs).	8
Table S12	Linear mixed model of log RBD - binding IgG and log NAbs including interaction between severity and Age group	9
Table S13	Linear mixed model of log RBD - binding IgG and log NAbs including interaction between severity and BMI group	11

Table S1 –Epidemiological and medical questionnaire

	Question	Answer 1	Answer 2	Answer 3	Answer 4
1.	What is your date of birth?				
2.	What is your gender?	Male	Female		
3.	What is your current height in meters?				
4.	What is your current weight in kg?				
5.	What is the exact date you received a positive PCR test for SARS-CoV-2?				
6.	During the COVID-19 infection did you experience any illness symptoms?	Yes	No		
7.	Did you experience a fever above 37.5? How many days did it last?	Yes	No	Number of days	
8.	Did you experience fatigue or weakness?	Yes	No		
9.	Did you experience muscle aches (myalgia)?	Yes	No		
10.	Did you experience a headache?	Yes	No		
11.	Did you experience reduced sense of test and smell?	Yes	No		
12.	Did you experience shortness of breath?	Yes	No		
13.	Did you experience cough?	Yes	No		
14.	Did you experience rhinorrhea?	Yes	No		
15.	Did you experience a sore throat?	Yes	No		
16.	Did you experience gastrointestinal inconvenience?	Yes	No		
17.	Did you require oxygen?	Yes	No		
18.	Did you require hospitalization?	Yes	No		
19.	Did you require intensive care?	Yes	No		
20.	Did you experience any other symptoms?	Describe			
21.	Do you take medications on a regular basis? If you do, what kind?	Yes	No	Specify	
22.	What was the date of the onset of symptoms?				
23.	Do you have any comorbidities?	0	1	2	3+
24.	Do you suffer from systemic hypertension (systolic blood pressure above 140) for which you are pharmaceutically treated?	Yes	No		

25.	Do you suffer from dyslipidemia (total cholesterol above 200 or LDL cholesterol above 160) for which you are pharmaceutically treated?	Yes	No		
26.	Do you suffer from an autoimmune disease for which you are pharmaceutically treated?	Yes	No		
27.	Do you have diabetes (HbA1C>6.5 or fasting blood sugar>126) for which you are pharmaceutically treated?	Yes	No		
28.	Do you suffer from heart disease for which you are pharmaceutically treated?	Yes	No		
29.	Do you suffer from lung diseases such as asthma, COPD, and pulmonary fibrosis for which you are pharmaceutically treated?	Yes	No		
30.	Do you suffer from any coagulation disorder resulting in hemorrhage or thrombosis for which you are pharmaceutically treated?	Yes	No		
31.	Are you immunosuppressed (organ transplant recipient, currently undergoing biologic therapy/chemotherapy, treated with corticosteroids, underwent a splenectomy, or diagnosed with HIV)?If yes, please state the specific condition	Yes	No		
32.	Have you ever had a serious allergic reaction (anaphylaxis) that required immediate treatment?	Yes	No		
33.	Do you have a liver disease as cirrhosis, hepatitis, liver cancer, or a metabolic disorder?	Yes	No		
34.	Do you have a kidney disease (a creatinine level of >1.2 mg/dL or GFR<60) for which you are pharmaceutically treated?	Yes	No		
35.	Are you currently pregnant (as confirmed by a beta-HCG blood test and fetal heartbeat detection on ultrasonography)?	Yes	No		

The questionnaire was reviewed and approved by the Institutional review board of the Sheba Medical Center.

Table S2- Medical follow-up questionnaire

	Question	Answer 1	Answer 2	Answer 3	Answer 4
1.	After the acute COVID-19 infection did you suffer from any post-COVID symptoms? For how long did you suffer?	Yes	No	Specify duration of symptoms	
2.	After the acute COVID-19 infection did you suffer from any mental post-COVID symptoms? For how long did you suffer from the mental symptoms?	Yes	No	Specify duration of symptoms	
3.	After the acute COVID-19 infection did you suffer from any neurological post-COVID symptoms? For how long did you suffer from the neurological symptoms?	Yes	No	Specify duration of symptoms	
4.	After the acute COVID-19 infection did you suffer from any cardiac post-COVID symptoms? For how long did you suffer from the cardiac symptoms?	Yes	No	Specify duration of symptoms	
5.	After the acute COVID-19 infection did you suffer from any pulmonary post-COVID symptoms? For how long did you suffer from the cardiac pulmonary?	Yes	No	Specify duration of symptoms	
6.	Have you visited a post-COVID clinic? If you did, when was the last time you visited the post-COVID clinic?	Yes	No	Specify date	

The questionnaire was reviewed and approved by the Institutional review board of the Sheba Medical Center.

Table S3: COVID-19 disease severity*

Severity of disease	Symptoms
Asymptomatic	No symptoms reported
Mild	Fever + other symptoms excluding: dyspnoea, cough, oxygen support, or other symptoms excluding: fever and dyspnea.
Moderate	Fever, cough, dyspnoea, fast breathing, but no signs of severe pneumonia, including SpO2 ≥ 90% on room air .
Sever	Fever, cough, dyspnoea, fast breathing, plus one of the following: respiratory rate > 30 breaths/min; severe respiratory distress; or SpO2 < 90% on room air.

*Taken from Clinical Management of COVID-19 WHO, Interim guidance, May 2020, page 13. World Health Organization (2020). Clinical management of COVID-19: interim guidance, 27 May 2020. World Health Organization. https://apps.who.int/iris/handle/10665/332196. License: CC BY-NC-SA 3.0 IGO

Table S4: Mixed Linear Models adjusted for sex and number of comorbidities, by period, for vaccinated versus recovered subjects (Outcome of log RBD - binding IgG)

Effect		Coefficient Estimate, (SE)	P value
Vaccinated - recovered	Vaccinated	1.08, (0.11)	<.0001
	Recovered	ref	
Period		-0.26, (0.01)	<.0001
period * vaccinated - recovered		-0.28, (0.01)	<.0001
Sex	1	0.11, (0.10)	0.30
	0	ref	
Number of comorbidities	1	0.20, (0.11)	0.08
	2+	0.24, (0.16)	0.14
	0	ref	

Table S5: Mixed Linear Models adjusted for sex and number of comorbidities, by period, for vaccinated versus recovered subjects (Outcome of log NAbs)

Effect		Coefficient Estimate, (SE)	P value
Vaccinated - recovered	Vaccinated	0.89, (0.14)	<.0001
	Recovered	ref	
Period		-0.12, (0.03)	<.0001
period * vaccinated - recovered		-0.17, (0.03)	<.0001
Sex	Male	0.25, (0.11)	0.02
	0	ref	
Number of comorbidities	1	0.22, (0.12)	0.06

	2+	0.14, (0.17)	0.38
	0	ref	

Table S6- Mixed linear regression model for calculating the difference between recovered and vaccinated subjects in two age groups (Outcome of log RBD - binding IgG)

Effect		Coefficient Estimate, (SE)	P value
Vaccinated vs recovered	Vaccinated	1.46, (0.14)	<.0001
	Recovered	ref	
Period		-0.26, (0.01)	<.0001
Age	>45	0.74, (0.17)	<.0001
	<45		
Sex	Male	0.09, (0.10)	0.40
	Female	ref	
Number of comorbidities	1	0.21, (0.11)	0.06
	2+	0.22, (0.16)	0.18
	0	ref	
period * vaccinated - recovered		-0.28, (0.01)	<.0001
Age (below and above 45 * vaccinated vs recovered)	1	-0.89, (0.19)	0.30
	0	ref	

Table S7- Mixed linear regression model for calculating the difference between recovered and vaccinated subjects in two BMI groups (Outcome of log RBD - binding IgG)

Effect		Coefficient Estimate, (SE)	P value
Vaccinated- recovered	Vaccinated	1.27, (0.12)	<.0001
	Recovered	ref	
Period		-0.26, (0.01)	<.0001
BMI recovered at peak	> 30	1.13, (0.23)	<.0001
	< 30	ref	
Sex	Male	0.17, (0.10)	0.11
	Female	ref	
Number of comorbidities	1	0.16, (0.11)	0.17
	2+	0.2, (0.16)	0.22
	0		
Period* Vaccinated-recovered		-0.28, (0.01)	<.0001
BMI (below and above 30 * vaccinated vs recovered)vaccinated at peak	1	-1.26, (0.26)	<.0001
	0	ref	

Table S8- Mixed linear regression model for calculating the difference between recovered and vaccinated subjects in females and males (Outcome of log RBD - binding IgG)

Effect		Coefficient Estimate, (SE)	P value
Vaccinated- recovered	Vaccinated	0.79, (0.18)	<.0001
	Recovered	ref	
Period		-0.26, (0.01)	<.0001
Sex	Male	-0.17, (0.17)	0.32
	Female	ref	
Number of comorbidities	1	0.19, (0.11)	0.10
	2+	0.24, (0.16)	0.13
	0		
Sex* Vaccinated- recovered	Male	0.43, (0.21)	0.05
	Female	ref	
Period * vaccinated vs recovered)		-0.28, (0.01)	<.0001

Table S9- Mixed linear regression model for calculating the difference between recovered and vaccinated subjects in two age groups (Outcome of log NAbs)

Effect		Coefficient Estimate, (SE)	P value
Vaccinated vs recovered	Vaccinated	1.26, (0.16)	<.0001
	Recovered	ref	
Period		-0.12, (0.03)	<.0001
Age	>45	0.43, (0.17)	0.01
	<45		
Sex	Male	0.22, (0.11)	0.04
	Female	ref	
Number of comorbidities	1	0.30, (0.12)	0.01
	2+	0.21, (0.17)	0.22
	0	ref	
period * vaccinated - recovered		-0.17, (0.03)	<.0001
Age (below and above 45 * vaccinated vs recovered)	1	-0.83, (0.19)	<.0001
	0	ref	

Table S10- Mixed linear regression model for calculating the difference between recovered and vaccinated subjects in two BMI groups (Outcome of log NAbs)

Effect		Coefficient Estimate, (SE)	P value
Vaccinated- recovered	Vaccinated	1.02, (0.15)	<.0001
	Recovered	ref	
Period		-0.12, (0.03)	<.0001
BMI	> 30	0.86, (0.24)	0.000
	< 30	ref	
Sex	Male	0.30, (0.11)	0.01
	Female	ref	
Number of comorbidities	1	0.20, (0.12)	0.09
	2+	0.12, (0.17)	0.46
	0	ref	
Period* Vaccinated- recovered		-0.17, (0.03)	<.0001
BMI (below and above 30 * vaccinated vs recovered)	1	-1.00, (0.26)	<.0001
	0	ref	

Table S11- Mixed linear regression model for calculating the difference between recovered and vaccinated subjects in females and males (Outcome of log NAbs)

Effect		Coefficient Estimate, (SE)	P value
Vaccinated- recovered	Vaccinated	0.54, (0.21)	0.01
	Recovered	ref	
Period		-0.12, (0.03)	<.0001
Sex	Male	-0.07, (0.17)	0.68
	Female	ref	
Number of comorbidities	1	0.20, (0.12)	0.08
	2+	0.16, (0.17)	0.34
	0		
Sex- Male vs Female* Vaccinated- recovered	1	0.51, (0.21)	0.02
	0	ref	
Period * vaccinated vs recovered)		-0.17, (0.03)	<.0001

Table S12 – Linear mixed model of log RBD - binding IgG and log NAbs including interaction between severity and age group.

Effect		Coefficient Estimate, (SE)	P value
**Log RBD - binding IgG**			
Period		-0.26, (0.02)	<.0001
Sex	Male	-0.13, (0.23)	0.57
	Female	ref	
Age	>45	0.73, (0.24)	0.003
	<45	ref	
Number of comorbidities	1	0.79, (0.33)	0.019
	2+	1.53, (0.52)	0.0043
	0	ref	
Severity level	Asymptomatic	2.48, (0.63)	0.0001
	Moderate	-0.08, (015)	0.6041
	Severe	0.1, (0.33)	0.7563
	Mild	ref	
Severity * Age	Asymptomatic* Age	-2.68, (0.89)	0.0033
	Asymptomatic* Age	ref	
	Moderate*Age	0.07, (0.32)	0.8318
	Moderate*Age	ref	
	Severe*age	0.00, (0.45)	0.993
	Severe*age	ref	

**Ratio of expected IgG of age>45 vs <45 in each severity level**				
	**Effect**	**Low CI**	**High CI**	**P value**
**Ratio of old vs young in asymptomatic severity level (peak IgG)**	0.14	0.03	0.79	0.0259
**Ratio of old vs young in mild severity level (peak IgG)**	2.08	1.29	3.35	0.003
**Ratio of old vs young in moderate severity level (peak IgG)**	2.22	1.07	4.60	0.0318
**Ratio of old vs young in severe severity level (peak IgG)**	2.08	0.80	5.43	0.1325

**Log NAbs**			
Period		-0.13, (0.03)	<.0001
Sex	Male	0.01, (0.21)	0.95
	Female	ref	
Age	>45	0.41, (0.23)	0.08
	<45	ref	
Number of comorbidities	1	0.64, (0.31)	0.04
	2+	0.87, (0.49)	0.08
	0	ref	
Severity level	Asymptomatic	1.22, (0.56)	0.03
	Moderate	0.1, (0.29)	0.74
	Severe	0.17, (0.72)	0.81
	Mild	ref	
Severity * Age	Asymptomatic* Age	-1.45, (0.82)	0.08
	Asymptomatic* Age	ref	
	Moderate*Age	0.58, (0.55)	0.29
	Moderate*Age	ref	
	Severe*age	-0.17, (0.89)	0.85
	Severe*age	ref	

**Ratio of expected NAbs of age>45 vs <45 in each severity level**				
	**Effect**	**Low CI**	**High CI**	**P value**
**Ratio of old vs young in asymptomatic severity level (peak NAbs))**	0.35	0.07	1.70	0.19
**Ratio of old vs young in mild severity level (peak NAbs)**	1.51	0.95	2.39	0.08
**Ratio of old vs young in moderate severity level (peak NAbs)**	2.69	0.93	7.73	0.07
**Ratio of old vs young in severe severity level (peak NAbs)**	1.27	0.22	7.26	0.79

Table S13 – Linear mixed model of log RBD - binding IgG and log NAbs including interaction between severity and BMI group.

Effect		Coefficient Estimate, (SE)	P value
**Log RBD - binding IgG**			
Period		-0.26, (0.02)	<.0001
Sex	Male	0.04, (0.24)	0.88
	Female	ref	
BMI	>30	0.86, (0.35)	0.01
	<30	ref	
Number of comorbidities	1	0.53, (0.36)	0.14
	2+	1.56, (0.54)	0.00
	0	ref	
Severity level	Asymptomatic	1.31, (0.46)	0.01
	Moderate	-0.12, (015)	0.40
	Severe	0.14, (0.23)	0.55
	Mild	ref	
Severity * BMI	Asymptomatic* BMI	0	
	Asymptomatic* BMI	ref	
	Moderate* BMI	0.27, (0.35)	0.44
	Moderate* BMI	ref	
	Severe* BMI	0.30, (1.39)	0.83
	Severe* BMI	ref	

**Ratio of expected IGG of BMI>30 vs <30 in each severity level**				
	**Effect**	**Low CI**	**High CI**	**P value**
**Ratio of >35 vs <35 in asymptomatic severity level (peak IgG))**	**Not estimated**			
**Ratio of >35 vs <35 in mild severity level (peak peak IgG)**	2.36	1.19	4.71	0.01
**Ratio of >35 vs <35 in moderate severity level (peak IgG)**	3.09	1.26	7.61	0.01

**Ratio of >35 vs <35 in severe severity level (peak IgG)**	3.19	0.21	48.91	0.40

Log NAbs			
Period		-0.13, (0.03)	<.0001
Sex	Male	0.12, (0.22)	0.59
	Female	ref	
BMI	>30	0.61, (0.32)	0.06
	<30	ref	
Number of comorbidities	1	0.40, (0.33)	0.22
	2+	0.95, (0.49)	0.05
	0	ref	
Severity level	Asymptomatic	0.66, (0.42)	0.11
	Moderate	0.18, (0.27)	0.49
	Severe	0.02, (0.44)	0.97
	Mild	ref	
Severity * BMI	Asymptomatic* BMI	0	
	Asymptomatic* BMI	0	
	Moderate* BMI	0.36, (0.66)	0.58
	Moderate* BMI	0	
	Severe* BMI	1.69, (1.54)	0.27
	Severe* BMI	0	

Ratio of expected NAbs of BMI>30 vs <30 in each severity level

	**Effect**	**Low CI**	**High CI**	**P value**
**Ratio of >35 vs <35 in asymptomatic severity level (peak NAbs))**	Not estimated			
**Ratio of >35 vs <35 in mild severity level (peak NAbs)**	1.84	0.97	3.48	0.06
**Ratio of >35 vs <35 in moderate severity level (peak NAbs)**	2.64	0.72	9.66	0.14
**Ratio of >35 vs <35 in severe severity level (peak NAbs)**	9.96	0.49	200.66	0.13
